# Reflex testing for Lynch syndrome: If we build it, will they come? Lessons learned from the uptake of clinical genetics services by individuals with newly diagnosed colorectal cancer (CRC)

**DOI:** 10.1007/s10689-013-9677-0

**Published:** 2013-09-04

**Authors:** E. Tomiak, A. Samson, N. Spector, M. Mackey, C. Gilpin, E. Smith, D. Jonker, J. Allanson, T. Asmis

**Affiliations:** 1 Department of Genetics, Eastern Ontario Regional Genetics Program, Children’s Hospital of Eastern Ontario, 401 Smyth Road, Ottawa, ON K1H 8L1 Canada; 2 Department of Medical Oncology, The Ottawa Hospital Cancer Centre, Ottawa, ON Canada; 3 The Ottawa Hospital Cancer Assessment Clinic, Ottawa, ON Canada; 4 Faculty of Education, University of Ottawa, Ottawa, ON Canada; 5 Faculty of Medicine, University of Ottawa, Ottawa, ON Canada

**Keywords:** Colorectal cancer, Lynch syndrome, Genetic counselling, Patient engagement, Universal screening, Health literacy

## Abstract

The aim of this qualitative study was to examine the experience of individuals facing a choice about genetic counselling/testing in the context of newly diagnosed colorectal cancer (CRC). Nineteen individuals with newly diagnosed CRC, including 12 individuals who accepted genetic counselling (“acceptors”) and 7 individuals who declined genetic counselling (“refusers”), were interviewed using a standardized questionnaire guide which focused on motivations and barriers experienced in the decision process. Data were analyzed using Karlsson’s Empirical Phenomenological method of data analysis (Karlsson in Psychological qualitative research from a phenomenological perspective. Almgvist and Wiksell International, Stockholm, 1993). Three major themes were identified: facing challenges in health literacy; mapping an unknown territory; and adjusting to cancer. The study participants’ testimonies provided novel insights into potential reasons for patient non-engagement in pilot studies of reflex testing for Lynch syndrome, and allowed us to formulate several recommendations for enhancing patient engagement. Our study findings suggest that patient engagement in clinical cancer genetics services, including reflex testing for Lynch syndrome, can only be achieved by addressing current health literacy issues, by deconstructing current misconceptions related to potential abuses of genetic information, by emphasizing the clinical utility of genetic assessment, and by adapting genetics practices to the specific context of cancer care.

## Introduction

Despite advances in our understanding of hereditary forms of colorectal cancer (CRC), the identification of individuals at high risk due to an inherited predisposition remains a significant clinical and public health challenge. The majority of individuals at high risk remain unidentified and uniformed about prevention and management strategies.

Factors affecting the uptake of clinical genetic services by individuals with suspected hereditary predisposition to CRC have included patient, health professional, and system variables [1]. Poor awareness of genetic testing for CRC predisposition has been identified among primary care physicians, gastroenterologists and oncologists [2–4]. Even in the setting of a well established North American gastroenterology cancer clinic, only a minority (17 %) of CRC patients eligible for genetic assessment were actually referred [4]. In the province of Ontario, only 28.3 % of Lynch syndrome (LS) cases identified in a provincial registry had been assessed by a clinical genetics service [5]. Similar data pertaining to under-recognition and under-referral have been reported in several American and European settings [6–8].

The existing literature has reported several patient factors associated with uptake of genetic counselling and testing for inherited cancer susceptibility [9]. In general, psychological factors have been identified to be more important determinants than socio demographic variables [9–14].

Most studies carried out to assess the uptake, motivations, and barriers to genetic testing for LS have been carried out in individuals already in the process of genetic testing [15] or in family members of individuals known to carry a LS mutation [11, 12, 14, 16]. There are few published reports of the experience of genetic counselling and testing in individuals recently diagnosed with CRC in whom a hereditary predisposition is suspected [17–19]. Despite the paucity of evidence concerning the acceptability of genetic testing in newly diagnosed CRC patients, experts recommend such testing in newly diagnosed CRC patients to reduce morbidity and mortality in relatives, and the implementation of universal screening for LS has been adopted as a 2020 developmental objective by the Office of Public Health Genomics in the United States [20, 21].

Reflex testing of colorectal tumours, defined as the routine screening of tumours for evidence of defective DNA mismatch repair as a phenotypic marker of LS, has been proposed as a strategy to increase identification of individuals with LS, and represents an emerging standard of care [22, 23]. Two cost effectiveness analyses of reflex testing in newly diagnosed CRC patients and a similar analysis in newly diagnosed endometrial cancer patients have demonstrated that cost effectiveness of such screening is comparable to accepted screening activities in the general population [24–26]. For several reasons, widespread adoption of reflex testing has proven to be a challenge in individual clinical centres, as well as in public health initiatives [27, 28]. One of the barriers identified in both the initial clinical experience and in a recent survey of US sites has been low uptake of genetic services by patients [22, 28]. In the initial Ohio State University experience, only 26.5 % of individuals with CRC identified to be appropriate for genetic assessment presented for counselling [28]. This same group reported that only 28 % of patients with endometrial cancer who would have been expected to benefit from genetic counselling were actually seen by a genetic counsellor [29]. From these data, it is clear that patient non-engagement represents a significant barrier to the potential effectiveness of reflex testing and that a more complete understanding of patient barriers to the uptake of clinical genetics services is required.

This study was designed to investigate the different factors affecting uptake of genetic counselling and testing in newly diagnosed CRC patients being seen in a Canadian academic hospital Cancer Assessment Clinic. The aim of this qualitative study was to examine the experience of individuals facing a choice about genetic counselling/testing in the context of newly diagnosed CRC, focusing on motivations and barriers encountered. A qualitative design allowed all aspects of the patient experience to emerge in the data. Although our study was carried out prior to the introduction of reflex testing, our findings have direct relevance for the identification of strategies to enhance patient engagement in clinical genetic services following an abnormal tumour screen in the context of reflex testing.

## Methods

### Study design

Since the focus of the present research was on the significance research participants ascribed to genetic counselling and testing, a qualitative methodology was chosen. This choice facilitates a holistic, inductive and naturalistic understanding of participants’ experiences. The Children’s Hospital of Eastern Ontario and the Ottawa Hospital Research Ethics Boards approved the study protocol.

### Participant selection and recruitment

Eligible participants were derived from the cohort of all newly diagnosed CRC patients (n = 332) presenting for an initial consultation at the Ottawa Hospital Cancer Assessment Clinic during the 9 month period of March through December 2010. Eligible patients were part of a risk assessment pilot study which addressed the role of an advanced practice nurse in identifying individuals appropriate for referral for genetics assessment. In this pilot study, 56/332 patients were identified to be high risk and eligible for genetic counselling. Forty-five patients were referred to Genetics and 32 were actually seen for counselling [30]. Eligible participants met Ontario provincial criteria for referral for Cancer Genetics Services [31].

Each participant was individually interviewed by the first (ET) or third author (NMPS) and asked to describe his/her experience of making a decision regarding genetic counselling using a written question guide. Interviews were audio-taped and later transcribed. Participants deemed unable to tolerate the interview or give informed consent were excluded.

Nineteen individuals from different families were recruited. Twelve of these individuals accepted to undergo genetic counselling (acceptors) and seven did not (refusers) (Table 1).

**Table 1:** Self-reported demographic characteristics

	Acceptors (n = 12)	Refusers (n = 7)
Female:male	5:7	2:5
Median time between diagnosis and interview (months)	5	3.5
Age range (median)	37–77 (59)	54–79 (62)
Children	11 (92 %)	4 (57 %)
Prior experience with genetic counselling	1 (8 %)	1 (14 %)
1st degree relatives with a cancer diagnosis	8 (66 %)	5 (71 %)
1st degree relatives deceased after cancer diagnosis	4 (33 %)	1 (14 %)
Post-secondary education	7 (58 %)	4 (57 %)
Worries effect daily mood	5 (42 %)	4 (57 %)

### Data analysis

The Empirical Phenomenological Psychological data analysis proposed by Karlsson [32] was used in this study. The process of analysis was divided into many stages. In these stages, the researcher aims to reach the highest level of abstraction possible to draw out the essential elements of the experience as perceived by participants. To enhance trustworthiness, the analysis was done by the three first authors using the consensual approach as described by Samson and Zerter [33].

## Results

Three main themes were identified. These themes are summarized in Table 2. Verbatim quotes demonstrating each category are included in the Appendix (Table 4).

**Table 2:** Themes identified

Theme	
I	Facing challenges in health literacy
II	Mapping an unknown territory
III	Adjusting to cancer

### Theme 1: Facing challenges in health literacy

The period preceding the personal diagnosis of CRC is characterized by health literacy challenges for both patients and health professionals. Patient challenges include ignorance of one’s family history, lack of knowledge regarding the implications of a family history of cancer, and more specifically, ignorance of the utility of genetic assessment. Patients perceive that these health literacy challenges are not adequately addressed by health professionals.

#### Patient lack of awareness

The participants have some awareness of their family history of cancer. This knowledge however is imprecise: specific details regarding the exact type of cancers, age at diagnosis and exact relationship of affected relatives may not be known.

According to participants, their knowledge is greatly influenced by family communication style and dynamics. In some families communication is sparse and there is significant ignorance about health matters including cancer. In other families there is better communication and a greater awareness of family history of CRC.

Prior to the diagnosis of cancer, there appears to be a clear disconnect between one’s family history of CRC and one’s own personal health history. Participants usually do not directly link the family history of cancer to their own personal risk. They tend to see family history as outside their own personal history. In fact, the family history and personal history are often perceived as two parallel entities rather than being more closely intertwined. Thus it is difficult for the individual to intuit a possible implication for his/her own future health.

Challenges with health literacy extend also to participants’ knowledge concerning genetics. Myths and misconceptions characterize participants’ understanding of genetics in general and genetic aspects of cancer predisposition. Knowledge about genetics is non-specific and difficult to relate to personal experience. In contrast to a participant’s knowledge of cancer, which is a familiar reality and part of daily existence, the realm of genetics is generally outside the tangible experience of most participants.

The vast majority of participants first heard about the possibility of genetic assessment when asked to participate in the current study. When asked to share their views on genetic assessment, some participants perceived it as synonymous with psychological counselling. For others, genetic assessment was related to unsubstantiated and sometimes bizarre beliefs about genetics and its intended use. In particular, for some participants, genetic assessment was linked to its eugenic aspects, or to other questionable uses of genetic information (for example risk stratification in the context of insurance).

Even when genetic assessment was perceived in a more positive way, it was usually not perceived to be of personal benefit/utility but as a contribution to broader scientific knowledge. Genetic assessment was also associated by some participants with the finding of a cure for cancer.

#### Health professionals’ passivity

In the context of ignorance of family history and genetic predisposition, participants are highly dependent on direct recommendations of their health care providers, both for referral to genetics services, and for specific cancer screening recommendations. According to our research, patients do not initiate more intensive screening because of the lack of an absolute recommendation of their health care provider. Unless actively encouraged by their care provider to seek genetic assessment or to undergo colonoscopy, participants will forego such screening based on a false sense of reassurance introduced by the lack of a specific recommendation.

In summary, patients were able to learn by themselves that CRC can sometimes be an inherited condition, however, there seemed for most participants to be an almost complete ignorance of genetic assessment. When there was an awareness of genetic assessment, in general, such assessment was perceived as negative. For the few participants who perceived genetic assessment in a positive way, the motivation was often misguided and associated with finding of a cure or providing researchers with an enhanced understanding of CRC causation. The combination of patient health care literacy challenges and health care professionals’ passivity leads to suboptimal uptake of genetics services and screening.

### Theme 2: Mapping an unknown territory

After the initial reaction to the cancer diagnosis, participants tried to make sense of what was happening to them. That effort included trying to identify a cause for the illness. Most of the participants readily identified that lifestyle factors could contribute to CRC, but most discounted their own lifestyles as a major predicting factor. It was easier for participants to find an answer in their family history. As much as the personal and family history were seen as two parallel stories, not intertwined, before the diagnosis, after the diagnosis that same family history became a new focus of investigation. Indeed, the diagnosis of CRC prompted an enquiry of a new nature. Participants started to explore more critically the genetic aspects of their family history. This search for understanding included reading, research and questioning of their health care providers.

The majority of participants heard for the first time about genetic assessment when asked to participate in the current research. When the process and aims of genetic assessment were described and explained, most expressed surprise that such a possibility could exist and that the possibility of genetic assessment had not been discussed with them by their physician. After being made aware of genetic assessment, participants expressed the opinion that they could have received more information and advice from their physician regarding genetic assessment.

Without a definite recommendation from their physician, participants were forced to make their own decision about proceeding with genetic counselling. Often this was not a fully informed decision, as the majority of patients were not provided with sufficient information and most did not have other resources to draw on such as a family member’s previous experience or public knowledge.

Specific contexts sometimes made decision-making more difficult. For example, some participants faced conflicting opinions from family members and when discouraged from genetic assessment, did not have enough knowledge to be able to argue against such advice in a well informed manner. This was particularly evident for individuals who were advised to avoid genetic assessment for fears related to insurability and privacy.

For many participants, after the process of genetic assessment was explained, the major motivation for agreeing to genetic assessment was to provide information for other family members. Participants who themselves had children were more likely to be interested in pursuing counselling and testing. Participants without children were more likely to decline assessment. No participants expressed that they were motivated to seek genetic assessment because of potential implications for themselves in terms of treatment or screening for other cancers.

In the process of becoming informed about genetic assessment, participants became much more aware of the possibility of stigmatization and the emerging implications for family members. Participants voiced clear concerns about access to and potential uses of their genetic information.

In summary, patients had to map an unknown territory related to genetic assessment. That process of mapping consisted of information seeking, a clarification and better understanding of their family history, and a careful consideration of the impact and clinical utility of genetic assessment on their family and on themselves.

### Theme 3: Adjusting to cancer

At the time that participants were offered the possibility of genetic assessment, most were actively engaged in the process of coping with a new cancer diagnosis. This included coming to terms with new physical limitations, as well as coping with the psychological and emotional aspects of cancer. Participants reported experiencing a wide spectrum of emotions including fear, anxiety, sadness, shock, disbelief and numbness. Participants were also concerned about the emotional impact that their cancer diagnosis was having on their relatives and close ones.

For many participants the knowledge that they were facing a severe health threat was a transformative event, forcing the individual to confront their own mortality, to question their identity, to redefine priorities, and to construct a new sense of normality. Many participants expressed uncertainties about their future and noted that the way they viewed their lives had markedly changed.

In the context of such major emotional and psychological adjustments, genetic counselling was generally not seen as a high priority.

For some participants the physical constraints imposed by the cancer diagnosis (fatigue, dealing with side effects, and need to attend other medical appointments), posed important barriers to accessing clinical genetics services. For participants who declined genetic assessment one of the major barriers was timing. Many patients felt that they did not have the personal resources to face yet another assessment/procedure. Having to set aside additional time or having to travel to another facility for counselling represented a major challenge.

In contrast, some participants cited convenience as a major reason that they agreed to counselling. These participants accepted counselling when offered in conjunction with their regular clinic appointments.

In summary, decision making regarding genetic counselling and testing was made in a very difficult situation characterized by invasive procedures, fear, uncertainty and vulnerability. Patients were confronting fears surrounding death and coping with the substantial physical demands and burden of cancer. For many participants, genetic assessment was not viewed as a high priority among many competing demands.

Just as importantly, there was no evidence that discussion of the genetic aspects was incorporated into the algorithm of care of newly diagnosed patients, nor was the integration of such knowledge acknowledged as one of the adaptive tasks faced by participants in adjustment to their diagnosis.

## Discussion

The inadequacy of physician initiated screening for LS has been well established, as has the heterogeneity in pathological assessment [34]. Multiple challenges to the implementation of universal screening for LS currently exist, including major infrastructural needs, and the recognition that not all of the patient and societal implications have been adequately addressed [27, 35]. Importantly, the success of reflex testing will be highly dependent on the acceptance of such testing by patients and their relatives, and on their compliance with enhanced screening [24–26].

The current study has provided three novel insights into potential reasons for patient non-engagement in pilot studies of reflex testing. Such insights may help to shape a framework for the successful implementation of population based screening for LS.

First, the current research indicates, from study participants’ perceptions, a need for educational interventions to improve health literacy for (1) The general public; (2) Patients diagnosed with colorectal or endometrial cancer; and (3) Primary care physicians and health care professionals providing specialized care for newly diagnosed CRC or endometrial cancer patients. These educational interventions should address:

- Lack of awareness regarding general genetic principles and lack of knowledge regarding the hereditary aspects of cancer.
- Lack of specific knowledge related to the clinical utility of genetic testing for LS in treatment planning and follow-up.
- Misinformation or lack of knowledge related to the use of testing results and the possibilities of insurance discrimination and lack of patient confidentiality.

Secondly, this study highlights the central importance of health care professional recommendations regarding genetic assessment. Without a specific discussion of the possible benefits (and harms) of genetic assessment, CRC patients are currently left with an impression that such assessment is not of value and are unlikely to pursue such assessment on their own initiative.

Thirdly, this study highlights the importance of recognizing the specific patient circumstances that exist at the time that genetic assessment is discussed, including emotional, physical and logistical aspects. Genetic counselling should provide compassionate individualized opportunities that are sensitive to the patient’s circumstance and needs. The data highlight the importance of provision of adequate psychosocial support and flexibility in timing of genetic assessment.

## Recommendations

Using these results we are able to generate recommendations targeted to three specific stakeholder groups (Table 3), which we believe will enhance patient engagement in reflex testing for LS.

**Table 3:** Recommendations for enhancing patient engagement in clinical genetic services, including reflex testing for Lynch syndrome

(A) General public level	Comprehensive education programs need to address current gaps in knowledge. Specifically the content of such programs needs to provide more objective and exact images of clinical genetic assessment, including specific information related to the utility of such testing in the treatment and prevention of CRC. Additionally it is imperative that current myths and misconceptions be targeted, as these continue to represent significant barriers preventing participation in testing.
(B) Individual CRC patient level	Each treating facility should include in their algorithm of care an appropriate assessment of family history, criteria for referral for genetic counselling/testing, and a discussion of genetic assessment tailored to patient need and knowledge. The treating facility should actively facilitate the process of genetic assessment, taking into account the context of illness, the readiness of the patient to undertake such an assessment, and the physical proximity of genetics services to usual place of care. Rather than assuming a standard optimal time for genetic assessment, the cancer system must adapt to the specific patient context, recognizing the low priority placed on genetic assessment by many patients.
(C) Health care professional level	Health care professionals need to recognize their central role in providing CRC patients with information and specific recommendation regarding genetic counselling/testing, and in encouraging appropriate cancer screening. Genetic considerations need to be addressed at the time of CRC diagnosis in a systematic way that is sensitive to patient needs and priorities, and in a way that includes concrete examples of the utility of genetic assessment in patient treatment plans and recommendations for family members. Multidisciplinary teams caring for CRC patients should be encouraged to incorporate genetic aspects into their algorithm of care for newly diagnosed CRC patients.

## Conclusion

The implementation of universal screening for LS has been adopted as a 2020 developmental objective by the Office of Public Health Genomics in the US and is currently being considered by other countries. Successful implementation of such programs will be highly dependent on achieving optimal patient engagement by addressing current gaps in health literacy, including the misconceptions related to potential abuses of genetic information, by inclusion of genetic assessment in the post-CRC diagnosis care map, and by adapting current clinical genetics services practices to the specific context of cancer care. We conclude that if we build it (with proper infrastructure using a public health approach), they may come, but only if the above challenges are addressed.

## Appendix

See Table 4.

**Table 4:** Verbatim quotes by theme

**Theme 1: Facing challenges in health literacy**
(A) Patient lack of awareness
It‘s not communicated within (families), you know everyone keeps to themselves, they don‘t really talk about illness a lot. (Acceptor, male, 37 years)
Yeah, for instance, this older cousin who had a … colon operation a year or two ago, never speaks to anybody about it. He didn‘t even speak to his own son about it. (Acceptor, male, 77 years)
I did know about it (colorectal cancer), but I never thought it would be, I thought it was mostly a male cancer. I never thought that, no, that I‘d be affected by it. Never even gave it a thought. (Acceptor, female, 57 years)
Genetic counselling, I‘ve never heard of. I don‘t know what and why, it almost has rings of it, for me, of sort of all those great genetic selection and those kind of issues. (Decliner, male, 58 years)
The testing then ends up getting in the hands of laboratories, drug companies. I read about a family or a group of families, I believe they were from New Brunswick, who have a… something special about their genetic traits. They were tested and now it turns out that some drug company owns the rights to their—well, I guess I don‘t agree with that, and so I wouldn‘t want to do anything that would have an effect on my family. (Decliner, female, 79 years)
It’s to help, in the process of finding a cure for cancer. (Decliner, male, 54 years)
(B) Health professionals’ passivity
And when he mentioned maybe a colonoscopy, we just sort of shrugged it off. But he never pushed it, I sort of felt that maybe if I had more pushing I would have done it. …I rolled the dice and I ignored the fact that I should be getting a colonoscopy… (Acceptor, female, 70 years)
They said we‘ll only go up to the turn in your bowel. But in that area they found 2, 3, or 4 polyps which they removed at the time. My family doctor always knew this; he was my principal care provider at the time. But in spite of this he didn't seem to feel that a colonoscopy was necessary (Acceptor,male, 77 years).
So I don‘t even know what‘s going to happen in this testing because not a shred of it‘s been explained…. you should really have the testing explained thoroughly in the beginning because it might influence somebody‘s choice. I don‘t know if it would have influenced my choice because I‘m here now going ahead with it. So, we‘ll see. (Acceptor, female 64 years)
**Theme 2: Mapping an unknown territory**
I don‘t actually think Dr. X. talked to me about that one. (Acceptor, female, 70 years)
And of course we talked to our children and nieces and nephews and nobody seemed to want to go and do it, they were afraid that it would be harder to get their life insurance and so on and so forth so. (Acceptor, female, 64 years)
I didn‘t see a result coming out of the counselling that would be helpful to me. (Decliner, male, 58 years)
It was about family, strictly. If I had children or grandchildren, both, I would have …very likely changed my opinion about whether or not to proceed with the counselling. (Decliner, male, 62 years)
No, it‘s not that I didn‘t want to do it (get the genetic testing), it‘s that I wanted to protect my kids. (Decliner, male, 54 years)
But that‘s not saying I would be willing to jeopardize my family….So, one of the most important factors is around privacy for your family…- I don‘t mind being poked and prodded and blood drawn and, if it‘s any help to anybody, but do I want to involve anything with my family? I think that decision is theirs and when they get a little older and if they run into problems, then maybe they will decide. (Decliner, female, 79 years)
I think there‘s a family up in Saguenay or something like that, who went through genetics. They have a disease all onto themselves and something like that. In that case, it‘s good for them to know this but it might have caused them a little bit of harm also... well it's just what everybody perceives them all of a sudden as, so they have to fight this sort of prejudice against them. (Acceptor,female, 57 years)
What are they going to do with it? And what‘s going to happen to it in 5 years, 10 years, 20 years? So… because there‘s nobody or no way that you really can say, hey, I want you to burn all that. And that‘s my concern with it… I think it‘s a good thing, but maybe it‘s the use that all this could be put to, that‘s my concern. (Decliner, female, 79 years)
**Theme 3: Adjusting to cancer**
If I considered it [genetic counselling] as being important, we probably would have worked on some sort of scheduling, but at that point in time, other things were on my mind and we were already on the road to having the operation done and that would be priority, you know. (Decliner, male, 67 years)
It wasn‘t really big on my list, I guess…. But I had to get chemo, I had to go for blood work, I didn‘t really have to do this. (Acceptor, female, 70 years)
Mmm… I‘m sort of busy right now…with something called cancer. (Decliner, male, 62 years)
My initial reaction when it was mentioned was put it in the drawer. I‘ve got just all I want to handle right now, let me get through this. (Decliner, female, 79 years)
Given that I got a lot on my plate, I‘m not interested. After this is over (laughs), if somebody came back to me and said, look, I understand you‘re reasonably well, you‘re over your surgery, you don‘t have any other real problems, any other big demands in your time or energy? No. Would you consider genetic testing because of these benefits to other people? Yeah, of course I would. (Decliner, male, 62 years)
I was going through an awful lot at that time, one more or one less didn‘t really matter. I live pretty far from the hospital so my biggest concern is to try and get everything packed into the same day, but sometimes it‘s pretty difficult to do that. (Acceptor, female, 57 years)
